# (–)-Epicatechin Provides Neuroprotection in Sodium Iodate-Induced Retinal Degeneration

**DOI:** 10.3389/fmed.2022.879901

**Published:** 2022-06-27

**Authors:** Manjuan Peng, Xuezhi Zhou, Fei Yao, Haibo Li, Weitao Song, Siqi Xiong, Xiaobo Xia

**Affiliations:** ^1^Eye Center of Xiangya Hospital, Central South University, Changsha, China; ^2^Hunan Key Laboratory of Ophthalmology, Xiangya Hospital, Central South University, Changsha, China; ^3^National Clinical Research Center for Geriatric Disorders, Xiangya Hospital, Central South University, Changsha, China

**Keywords:** age-related macular degeneration, (–)-epicatechin, mitochondrial dynamics, mitochondrial quality control, TMEM97, amyloid beta

## Abstract

Oxidative stress, mitochondrial impairment, and pathological amyloid beta (Aβ) deposition are involved in the pathogenesis of dry age-related macular degeneration (AMD). The natural flavonoid (–)-epicatechin (EC) is known to be an antioxidant and neuroprotective compound. Whether EC plays a therapeutic role in AMD is unknown. In this work, we aimed to assess the efficacy and molecular mechanisms of EC against sodium iodate (NaIO_3_)-induced retinal degeneration in C57BL/6 mice *via* bioinformatic, morphological, and functional methods. We demonstrated that EC had no toxic effects on the retina and could ameliorate retinal deformation and thinning. EC treatment prevented outer retinal degeneration, reduced drusen-like deposits, increased b-wave amplitude in electroretinography, blocked retinal gliosis, and increased the number and quality of mitochondria. Importantly, EC increased the protein expression of OPA1 and decreased the expression of PINK1, indicating the role of EC in mitochondrial fusion that impaired by NaIO_3_. Moreover, EC downregulated APP and TMEM97 levels, upregulated PGRMC1 levels, and reduced subretinal Aβ accumulation. This study illustrated that EC, which may become a promising therapeutic strategy for AMD, prevented NaIO_3_-induced retinal degeneration, and this improvement may be associated with the mitochondrial quality control and the TMEM97/PGRMC1/Aβ signaling pathway.

## Introduction

AMD accounts for approximately 5.8% of legal blindness globally and is the leading cause of severe vision loss in the developed world (1). It is estimated that ~288 million people will suffer from this blindness-causing eye disease by 2040 (2). Currently, while laser and anti-vascular endothelial growth factor therapies are widely used to treat wet AMD, an effective treatment for dry AMD is still lacking.

Oxidative stress, mitochondrial dysfunction, and drusen deposits are major pathological factors triggering AMD (1, 3). Disrupted mitochondrial morphology, decreased mitochondrial number, and impaired mitochondrial dynamics have been found in the retinas of AMD donors and AMD mice (4, 5). Compounds that improve mitochondrial function serve as attractive therapies against AMD (6).

A growing body of evidence has suggested that retinas with AMD develop an Alzheimer's disease-like pathology, which is the amyloid beta (Aβ) peptide deposited within drusen (7). Anti-Aβ therapies could preserve the integrity of photoreceptor cells (8), block histopathologic changes, and attenuate declines in visual function (9), indicating that Aβ-related signaling pathways may be important therapeutic targets for AMD (10).

The potential of flavonoids to prevent mitochondrial dysfunction, protect neurons against oxidative stress, reduce the aggregation of Aβ protein, and modulate cell signaling pathways was recently highlighted (11).

(–)-Epicatechin (EC) is one of the most abundant flavonoids and is present in fruits, cocoa, and green tea (12). Several studies, most of which focused on the heart, muscles, and brain, have reported the relationships of EC supplementation with mitochondrial improvements. These studies found that exogenous EC could increase mitochondrial content, improve mitochondrial function, upregulate key antioxidant systems, and activate the central transcription factors of mitochondrial biogenesis (13–15).

Moreover, EC was shown to prevent Aβ-induced neuronal cell death in cell lines (16) and to decrease Aβ-induced lipid peroxidation, astrocytosis, and reactive oxygen species formation in the hippocampus of rats (17, 18). EC also exerted a protective action on learning and memory skills (17, 18), indicating its effective potential for the treatment of age-associated neurodegenerative disease. However, few studies have focused on the role of EC in retinal degenerative diseases, and the mechanisms underlying EC-associated restorations remain unclear.

Thus, we planned to determine differentially expressed genes (DEGs) and identify enriched pathways in human AMD samples to provide us with insight into the pathological mechanisms of AMD. We also aimed to explore the beneficial effects of EC on retinal morphology and function, as well as the loss of mitochondria, in a sodium iodate (NaIO_3_)-induced AMD mouse model.

## Materials and Methods

### Animals

This study was approved by the Animal Ethics Committee of Central South University (NO. 2019sydw0195). All experiments were conducted in accordance with the criteria of the *National Institutes of Health Guide for the Care and Use of Laboratory Animals*. Male C57BL/6 mice aged 6–8 weeks (weight, 18–22 g) were purchased from Hunan SJA Laboratory Animal Co., Ltd. (Changsha, China). Mice were maintained in individual ventilated Plexiglas cages under a 12 h light/dark cycle at room temperature in the Department of Laboratory Animals of Central South University throughout the experiment.

### Experimental Groups and Treatments

NaIO_3_ (40 mg/kg) (Sigma-Aldrich, St. Louis, MO, USA) and EC (100 mg/kg/day) (Sigma-Aldrich, St. Louis, MO, USA) were dissolved in sterile normal saline and sterile drinking water, respectively. The concentrations of drugs were chosen according to the body of literature (19, 20).

To evaluate the safety of EC, mice were randomly allocated into one of two groups: (1) the control group, which received only sterile drinking water by oral gavage, and (2) the EC group (at a dose of 100 mg/kg/day, by oral gavage). To assess the efficacy of EC, mice in the control group were given tail vein injections containing only 10 mL/kg normal saline; mice in the NaIO_3_ or EC group were treated with drinking water or 100 mg/kg/day EC orally *via* gastric gavage respectively, and given 40 mg/kg NaIO_3_ by tail vein injection half an hour after gastric gavage. Gavage administration of water or EC was given once daily for 7 days, and subsequent experiments were then carried out.

### Histopathology

Mice were sacrificed and transcardially perfused with 10 mL pre-cooled normal saline. The eyes were enucleated and fixed in FAS eye fixation fluid (Servicebio, Wuhan, China) for 24 h. Next, they were gradient dehydrated and embedded in paraffin. The paraffin eyes were sliced into 3 μm sections on the sagittal plane through the optic nerve (Leica, Wetzlar, Germany). Hematoxylin and eosin (HE) staining was carried out using an HE staining kit (Servicebio, Wuhan, China).

Light microscopy (Leica, Wetzlar, Germany) was used to obtain retinal images located 200–300 μm from the optic nerve head, which were compared for morphological observation and nuclei counting in the outer nuclear layer (ONL). Using Image Pro Plus 6.0, four images were grabbed at ± 250 and ± 500 μm per eye for quantification of the total area of drusen-like deposits. Also analyzed were the average thickness and the thickness at ± 150, ± 300, ± 450, ± 600, ± 750, ± 900, ± 1,050, ± 1,200, ± 1,350, ± 1,500 μm from the optic nerve of the ONL. The IS/OS were also analyzed.

### Fundus Photography and Optical Coherence Tomography Examination

After 7 days of oral gavage of water or EC, mice were anesthetized by an intraperitoneal injection of 0.2 ml/ 20 g of 1.0% pentobarbital sodium (Merck, Darmstadt, Germany). Pupils were dilated with one drop of 0.5% compound tropicamide (Santen, Tokyo, Japan) 5 mins before examination. Hydroxypropyl methylcellulose ophthalmic demulcent solution was applied to the corneal surface to form a uniform optical interface. Ocular fundus and OCT images were captured using a Micron IV retinal imaging system and Reveal OCT2 imaging system (Phoenix Research Labs, Pleasanton, CA, USA), respectively.

### Quantification of Retinal Degeneration From Color Fundus Photographs

To measure the percentage of retinal degeneration area, color fundus images were converted to gray scale 16-bit images and threshold using Image Pro Plus 6.0. Region of degeneration was then determined. The area of optic disc was substracted, and ratio of degeneration area/total area was calculated.

### Electroretinography

Following 8–12 h pre-adaptation to darkness, the mice were anesthetized, and the pupils were maximally dilated under red light. Full-field ERGs were recorded according to the International Society for Clinical Electrophysiology of Vision standards using a RETIport system (Roland Consult, Brandenburg, Germany) (21). The dark-adapted 3.0 ERGs were handled in a typical manner, with both a-wave and b-wave amplitudes analyzed.

### Bioinformatics Analyses

Gene expression data (.tsv files) from postmortem eyes were downloaded from the GEO database (GSE135092), which compromising 128 macular retinal samples. Donors whose age were unknown were excluded for further analysis. A total of 57 normal control and nine AMD macular retinal samples from donors ranging from 75 to 90 years of age were included in the differential expression analysis. DEGs were identified with the DESeq2 package in R 4.1.2 and then ranked based on the simple *p*-values (<0.05). Gene ontology (GO) enrichment analysis and Gene set enrichment analysis (GSEA) were performed using the clusterProfiler and GGplot2 R package.

### Immunofluorescence

The paraffin-embedded retinal tissue sections were dewaxed to water, immersed in citric acid antigen repair buffer, boiled for antigen repair, and blocked with 10% goat serum (Beyotime, Jiangsu, China) for 2 h. Then the sections were incubated at 4 °C overnight with antibodies against glial fibrillary acidic protein (GFAP) (1:200; CST; #12,389), glutamine synthetase (GS) (1:250; Abcam; ab178422), and Aβ (1:200; Biolegend; #805,509).

### Transmission Electron Microscopy

Mice were sacrificed by cranio-cervical dislocation. Eyes were immediately enucleated and fixed in a sufficient amount of precooled 2.5% glutaraldehyde fixing solution (Servicebio, Wuhan, China) for 2 h. The anterior segments were removed, and eyecoat at the posterior pole was cut into square pieces measuring approximately 1 to 2 mm^2^. After postfixing with 1% osmium tetroxide, dehydrating with graded acetone, embedding with resin, and slicing with an ultramicrotome (Leica Microsystems, Wetzlar, Germany), thin sections (50–100 nm) were examined on a HT7700 TEM (Hitachi, Tokyo, Japan).

### Western Blot Analysis

Mice were sacrificed, and eyes were quickly enucleated. The cornea, iris, lens, and vitreous body were removed from the eye in cold PBS solution under a dissecting microscope (Olympus, Tokyo, Japan). Fresh retinas were lysed in RIPA buffer (NCM Biotech Co., Ltd., Suzhou, China) containing a protease inhibitor cocktail (APExBIO, Houston, TX, USA). The retinas were then ground with a cryogenic tissue grinder (Servicebio, Wuhan, China). After determination of the concentration of protein in each group using a BCA protein assay kit (Pierce; Thermo-Fisher Scientific, Waltham, MA, USA), the lysates were boiled. Proteins in the lysates were separated by 12% sodium dodecyl sulfate–polyacrylamide gel electrophoresis and subsequently transferred to PVDF membranes (EMD Millipore, Burlington, MA, USA). The membranes were blocked with 5% bovine serum albumin (NeoFroxx GmbH, Einhausen, Germany) for 1.5 h at room temperature and incubated overnight at 4 °C with primary antibodies. Antibodies against optic atrophy 1 (OPA1) (1:1 000; #80,471), dynamin-related protein 1 (DRP1) (1:1 000; #8,570), mitofusin 2 (MFN2) (1:1 000; #9,482), amyloid precursor protein (APP) (1:1 000; #76,600), and progesterone receptor membrane component 1 (PGRMC1) (1:1 000; #13,856) were obtained from Cell Signaling Technology (CST, Beverly, MA, USA). Antibodies against PTEN-induced kinase 1 (PINK1) (1:300; sc-517353) were obtained from Santa Cruz Biotechnology (Santa Cruz, CA, USA). Antibodies against transmembrane Protein 97 (TMEM97) (1:500; nbp1-30437) was purchased from Novus (Novus, St. Louis, MO, USA). An antibody against β-actin (1:2000; 66009-1-Ig) was purchased from Proteintech (Wuhan, China) and used as an internal control.

### Statistical Analysis

All statistical analyses were performed using GraphPad Prism 7.0 (GraphPad Software, CA, USA). For comparison between two groups, Student's *t*-test was used; for comparison between multiple groups, one-way ANOVA followed by Bonferroni's multiple comparison test were used. Data are presented as mean ± SEM. *P*-values < 0.05 were considered statistically significant.

## Results

### EC Did Not Cause Retinal Morphology or Structural Impairment

**Figure 1:**
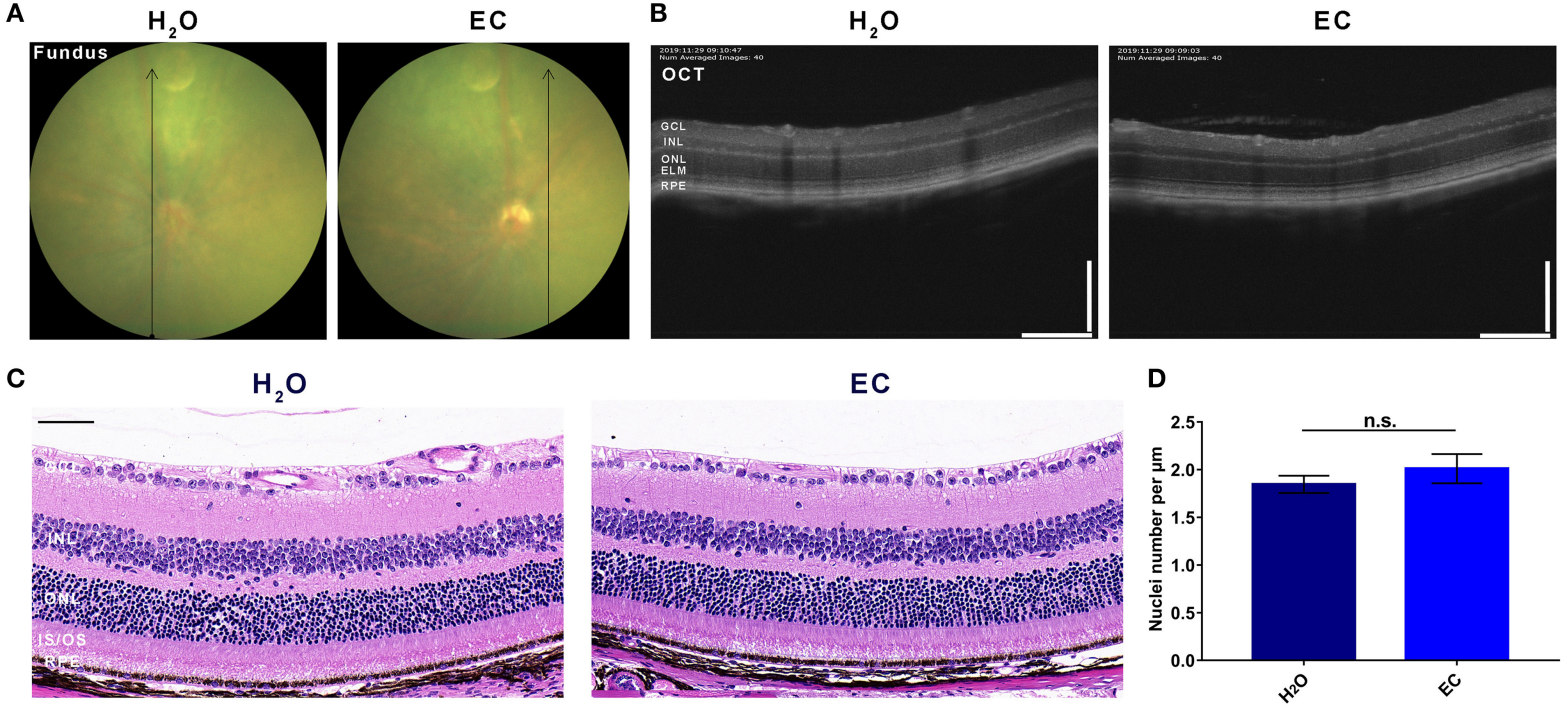
Safety validation of EC on the retina of C57BL/6 mice. Representative images of **(A)** fundus photography, **(B)** OCT, and **(C)** HE stained sections show no lesions in the EC group, and there is no observable structural change between the two groups. **(D)** Quantification of the number of nuclei per μm in the ONL. GCL, Ganglion cell layer; INL, inner nuclear layer; ONL, outer nuclear layer; ELM, external limiting membrane; IS/OS, inner segment/outer segment; RPE, retinal pigment epithelium. Values are presented as the mean with SEM, *n* = 3; n.s., no significance. Scale bar: 50 μm.

Compared to drinking water, 100 mg/kg/day EC had no apparent toxic effects on the retina by day 7 (Figure 1). Fundus photography did not reveal any observable lesions in the EC group (Figure 1A). According to OCT (Figure 1B) or the HE-stained sections (Figure 1C), the oral EC treatment did not cause morphological changes in the retina. The average number of nuclei in the ONL was not significantly different between the two groups (Figure 1D). These results indicate that oral administration of EC is safe for retinal use in C57BL/6 mice.

### EC Protected Against NaIO_3_-Induced Retinal Degeneration

**Figure 2:**
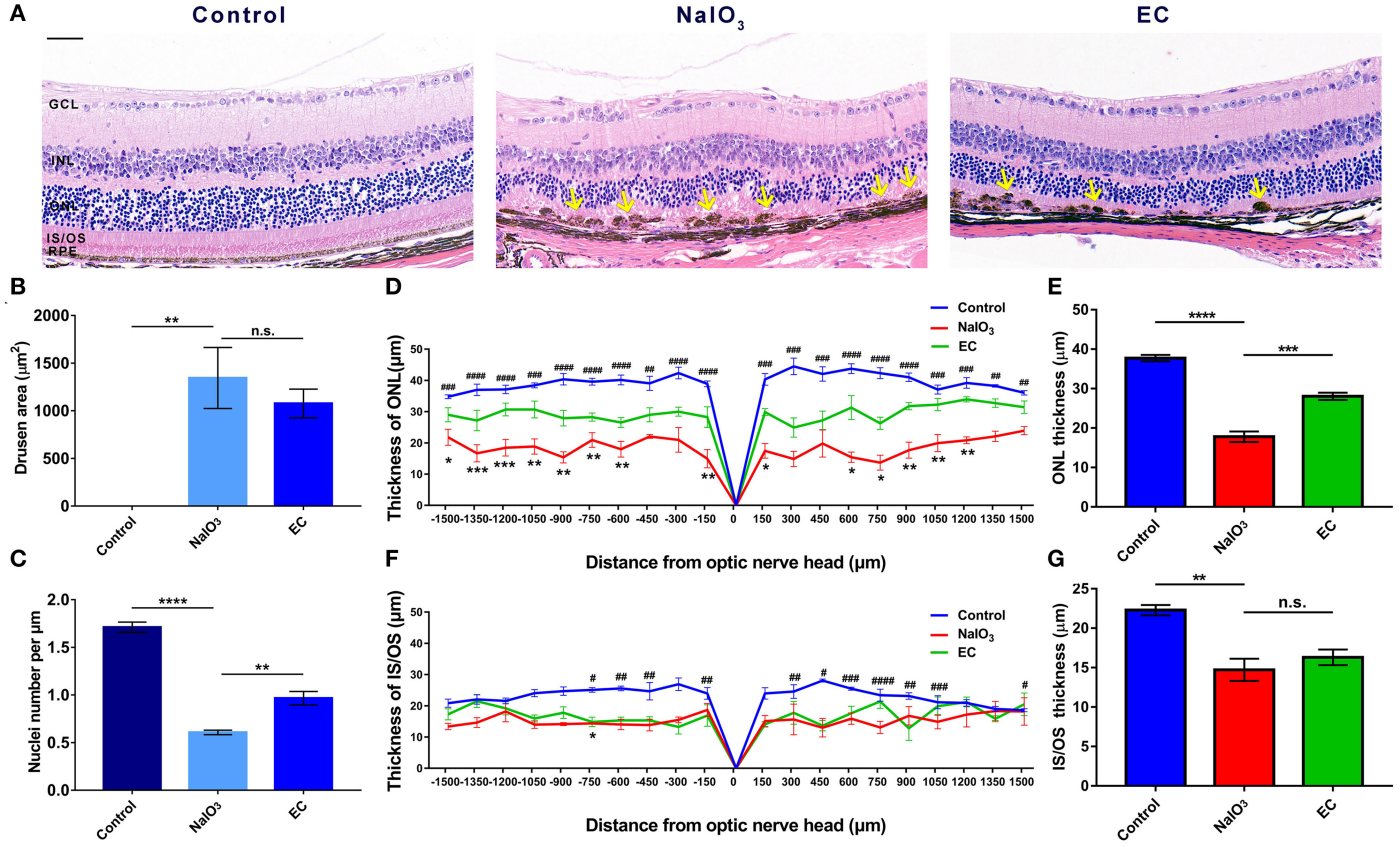
EC protects against retinal degeneration caused by NaIO_3_ administration. **(A)** The ONL underwent deformation by day 7 after NaIO3 administration, but this damage was ameliorated by EC treatment. Yellow arrows indicate drusen-like melanin deposits. **(B)** The total area of drusen-like deposits tended to be smaller and reduced in the EC group. **(C)** The number of nuclei per μm in the ONL was significantly increased in the EC group. **(D)** Thickness of the ONL and **(F)** IS/OS layers were measured at 150 μm intervals from the optic nerve head (#, Control vs. NaIO_3_ group; *, NaIO_3_ vs. EC group). **(E)** Mean thickness of the ONL and **(G)** IS/OS layers. GCL, Ganglion cell layer; INL, inner nuclear layer; ONL, outer nuclear layer; IS/OS, inner segment/outer segment; RPE, retinal pigment epithelium. Values are presented as mean with SEM, *n* = 4; n.s., no significance; *p < 0.05, **p < 0.01, ***p < 0.001, ****p < 0.0001. Scale bar: 50 μm.

To explore the retinal protective effects of EC, we established a mouse model of NaIO_3_-induced retinal degeneration mimicking dry AMD by intravenous injection of 40 mg/kg NaIO_3_. NaIO_3_ caused disorganization of photoreceptors and sharp retinal thinning, especially in the ONL, by day 7. EC apparently ameliorated deformation of the outer retina (Figure 2A), partly decreased the total area of drusen-like deposits (although the trend was not statistically significant) (Figure 2B), increased the number of photoreceptor nuclei in the ONL (Figure 2C), and protected the ONL from severe NaIO_3_-induced thinning (Figures 2D,E). However, differences between the NaIO_3_ group and the EC group in the thickness of the IS/OS layer were not statistically significant (Figures 2F,G).

### EC Reduced Drusen-Like Deposits and Outer Retinal Degeneration

**Figure 3:**
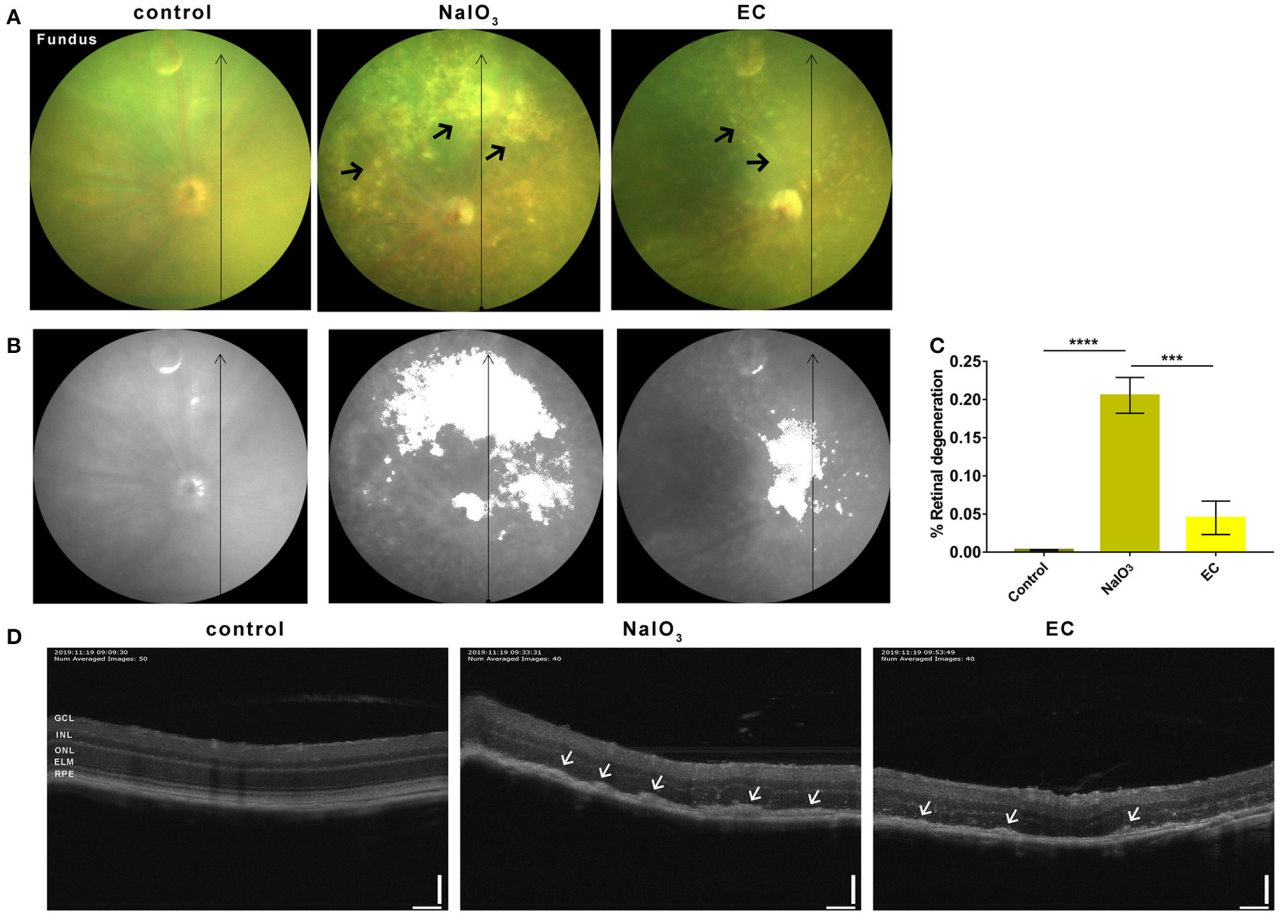
EC reduced outer retinal degeneration induced by NaIO_3_ administration. **(A)** Representative color fundus photographs taken at day 7 post-NaIO_3_ administration showed that EC reduced the number of yellow-white deposits. Black arrows indicate drusen-like deposits. **(B)** Color fundus photographs were converted to gray scale with retinal degeneration areas show in white. **(C)** The percentage of retinal degeneration (degeneration area/total area) were quantified. **(D)** Representative OCT images showed EC reduced hyperreflective foci in the outer retinal layers. White arrows indicate high reflex lesions. GCL, Ganglion cell layer; INL, inner nuclear layer; ONL, outer nuclear layer; ELM, external limiting membrane; RPE, retinal pigment epithelium. Values are presented as mean with SEM; *n* = 4 or 5. ^***^p < 0.001, ^****^p < 0.0001; Scale bar: 50 μm.

To further confirm the effectiveness of EC on a degenerative retina *in vivo*, we conducted non-invasive eye examinations. Color fundus photographs intuitively showed that exposure to NaIO_3_ could induce diffuse dot-like foci or patchy yellow-white lesions in the retina, and oral gavage of EC significantly reduced those drusen-like deposits on the fundus (Figure 3A). The areas of degeneration were sharply reduced when used EC (Figures 3B,C). OCT images revealed that the outer retinal layers in eyes treated with systemic NaIO_3_ were unclear, disorganized, and hyper reflective, but the hyper reflective foci were reduced when treated with EC (Figure 3D).

### EC Partially Rescued Retinal Function Impaired by NaIO_3_ Administration

**Figure 4:**
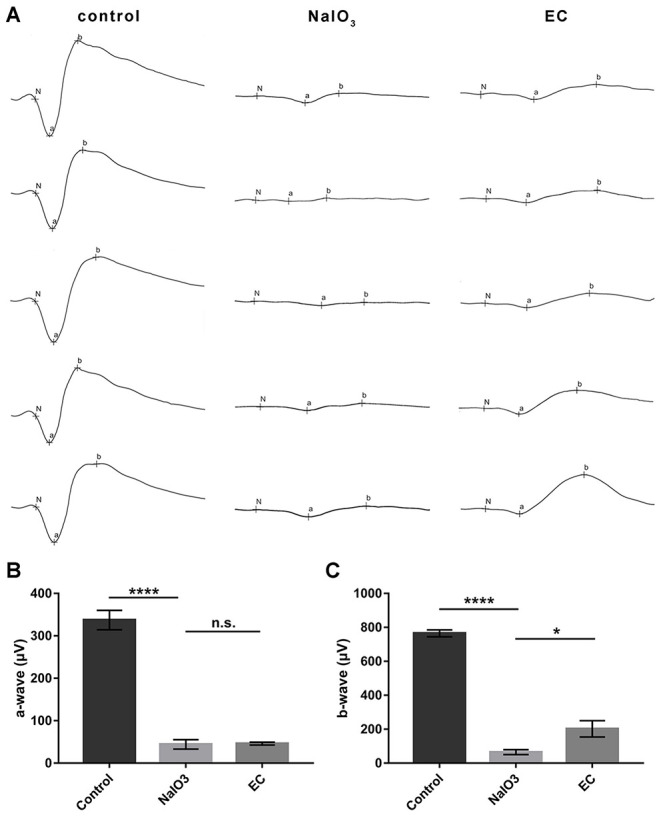
EC partially rescued retinal function impaired by NaIO_3_ administration. **(A)** Dark-adapted 3.0 ERGs of full-field ERG in the control, NaIO_3_, and EC groups. **(B)** A steep decline of a-waves was seen after NaIO_3_ injection, EC intervention did not exhibit significant benefits on a-wave amplitude. **(C)** EC exerted marked recovery effects on b-wave amplitude. Values are presented as mean with SEM, *n* = 5 or 6; n.s., no significance, *p < 0.05, ****p < 0.0001.

In addition to the effects on retinal morphology and structure, we then assessed how EC works on visual function. Dark-adapted 3.0 ERG showed that flat responses with both the a- and b-wave amplitudes were substantially decreased, by nearly 90% after the intravenous injection of NaIO_3_ (Figure 4A). This indicates that combined responses arising from photoreceptors and second-order neurons were severely impaired. Although the a-wave amplitudes, which are physiologically generated from photoreceptors, did not show statistically significant changes with EC intervention (Figure 4B), an approximate 130 μV increase in b-wave amplitude was observed with the recovery effects of EC (Figure 4C).

### Bioinformatics Analyses of Transcriptome Data in Human AMD

**Figure 5:**
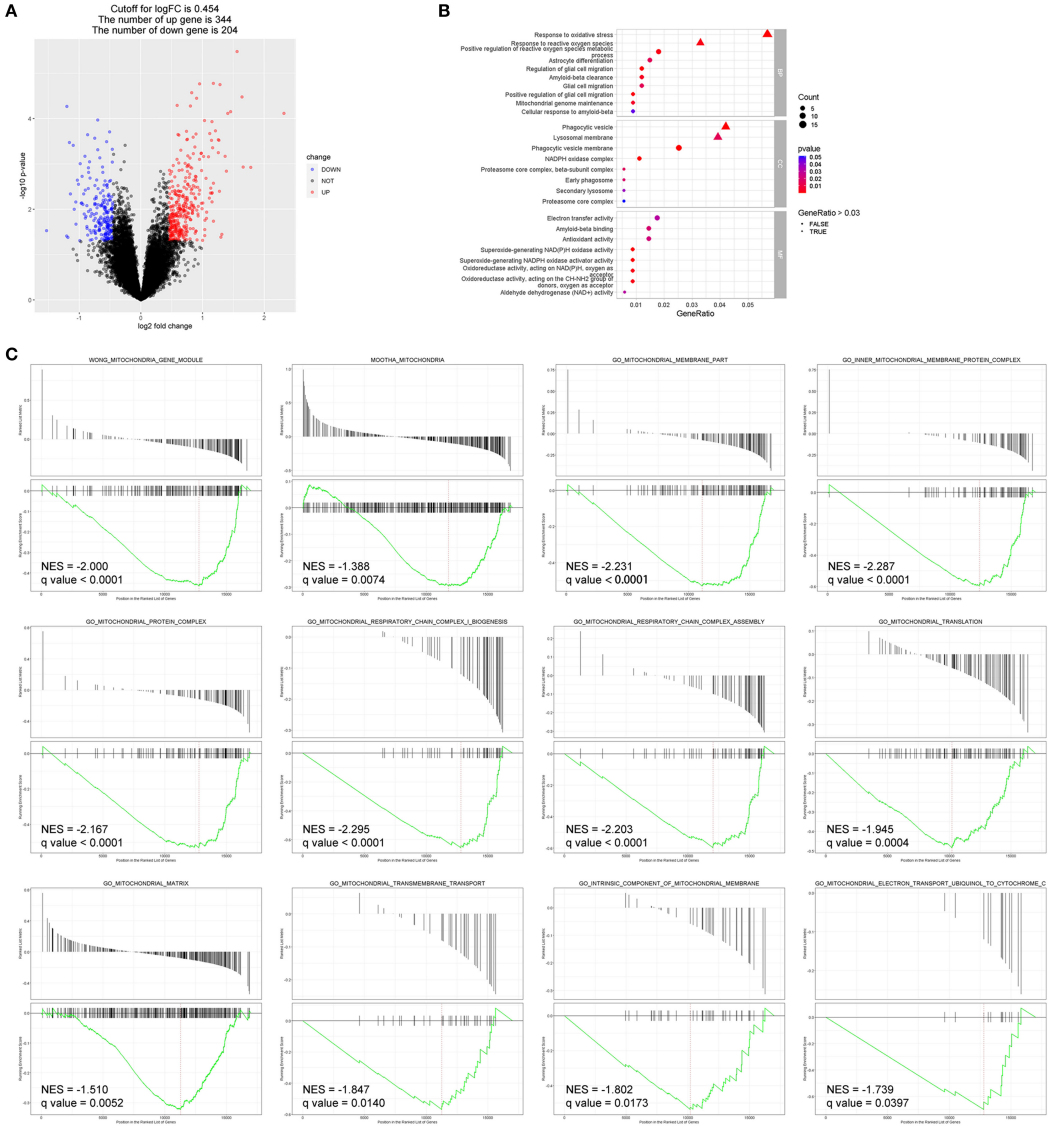
Bioinformatics analyses identified DEGs and enriched pathways in AMD (GEO: GSE135092). **(A)** The DEGs between the two groups are displayed by volcano map, including 344 upregulated genes and 204 downregulated genes (*p* < 0.05). **(B)** Enriched GO terms of interest were displayed after GO enrichment analysis of DEGs (*p* < 0.05). **(C)** GSEA of the mitochondria-related gene sets enriched in AMD retinas as compared to control retinas (*q*-value < 0.05). BP, biological process; CC, cellular component; MF, molecular function; NES, normalized enrichment score.

To better explain molecular mechanisms underlying the protective effects of EC, we then identified key genes and pathways associated with AMD using gene expression profiling data of human retina samples (GSE135092). Compared to the normal controls, 548 DEGs in AMD samples were identified, with 344 genes upregulated and 204 genes downregulated (Figure 5A, Data S1). Among the top 100 genes with the most obvious expression changes (Data S2), TMEM97 (log_2_FC = −0.637, *p* = 0.0079) and VTN (log_2_FC = −0.502, *p* = 0.0491) are associated with AMD (22).

Enriched GO terms of interest are displayed in Figure 5B: DEGs were significantly enriched in biological processes such as response to oxidative stress (GO: 0006979), response to reactive oxygen species (GO: 0000302), astrocyte differentiation (GO: 0048708), glial cell migration (GO: 0008347), and Aβ clearance (GO: 0097242); and in molecular function such as Aβ binding (GO: 0001540) and antioxidant activity (GO: 0016209).

Additionally, DEGs that significantly enriched in pathways related to mitochondrial structure and function showed reduced expression in the AMD group compared to the control group in GSEA (Figure 5C).

### EC Blocked Retinal Gliosis

**Figure 6:**
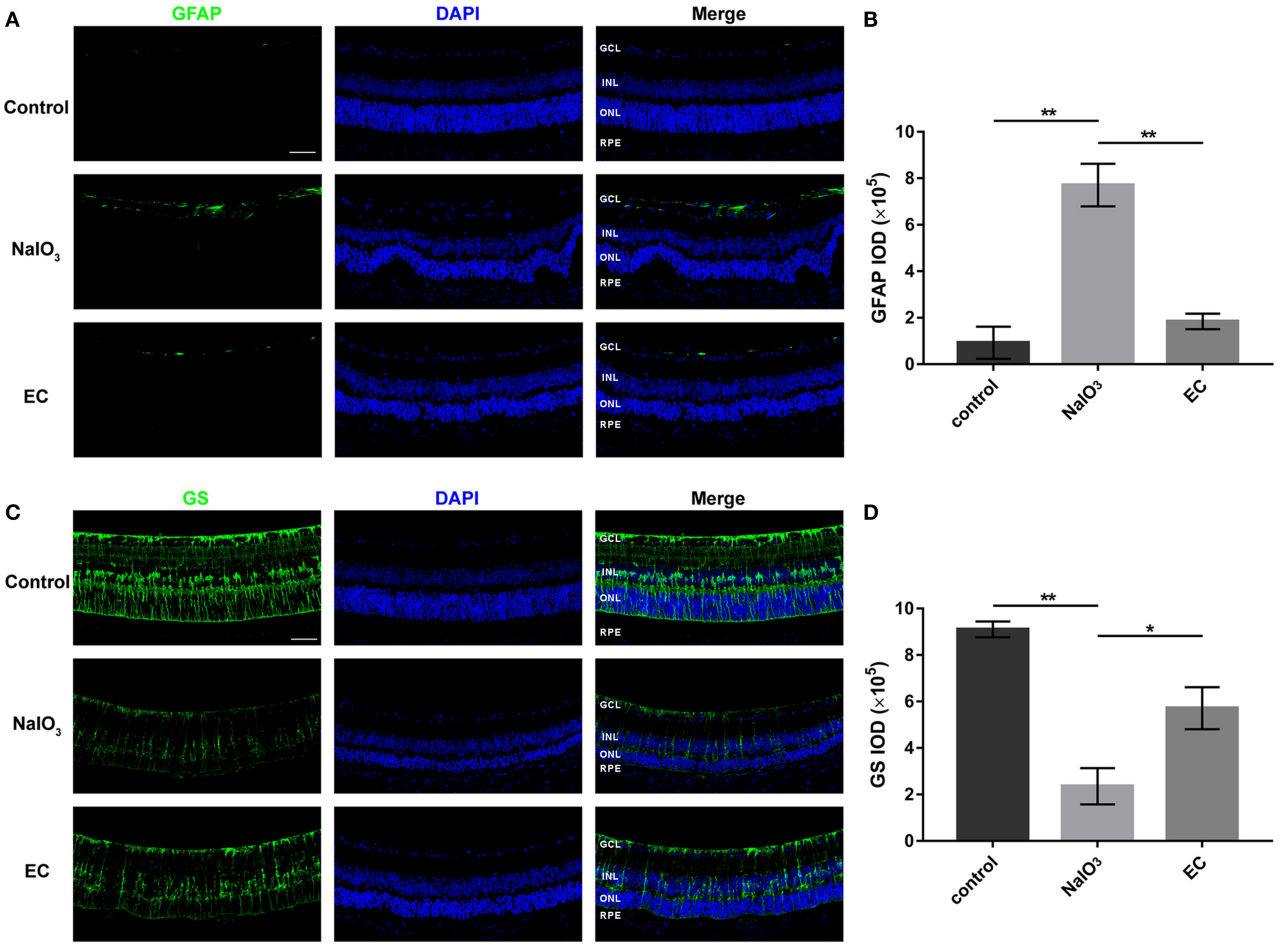
EC reduced astrogliosis and protected Müller cells in NaIO_3_-treated retinas. **(A)** Immunofluorescence images show GFAP intensity in the three groups. GFAP (green), DAPI (blue). **(B)** Quantification of GFAP fluorescence intensity. **(C)** Immunofluorescence images show GS intensity in the three groups. GS (green), DAPI (blue). **(D)** Quantification of GS fluorescence intensity. GCL, Ganglion cell layer; INL, inner nuclear layer; ONL, outer nuclear layer; RPE, retinal pigment epithelium; IOD, integrated optical density. Values are presented as the mean with SEM; *n* = 3. *p < 0.05, **p < 0.01.

Astrocyte and Müller cells regulate GFAP and GS expression in response to retinal injuries (23). Since genes involved in glial cell migration and astrocyte differentiation were enriched in GO analysis in AMD retinal samples, we studied retinal gliosis using GFAP and GS immunostaining in NaIO_3_-induced retinal degenerative mice. Figure 6 shows that GFAP levels markedly increased with NaIO_3_ administration, as expected. This enhancement was reduced by the EC administration (Figures 6A,B). The intensity of GS staining was sharply reduced in the NaIO_3_-treated retinas as compared to the control retinas, but subsequent EC treatment showed an enhancement (Figures 6C,D).

### EC Reduced Mitochondria Loss and Maintained Mitochondrial Morphologies

**Figure 7:**
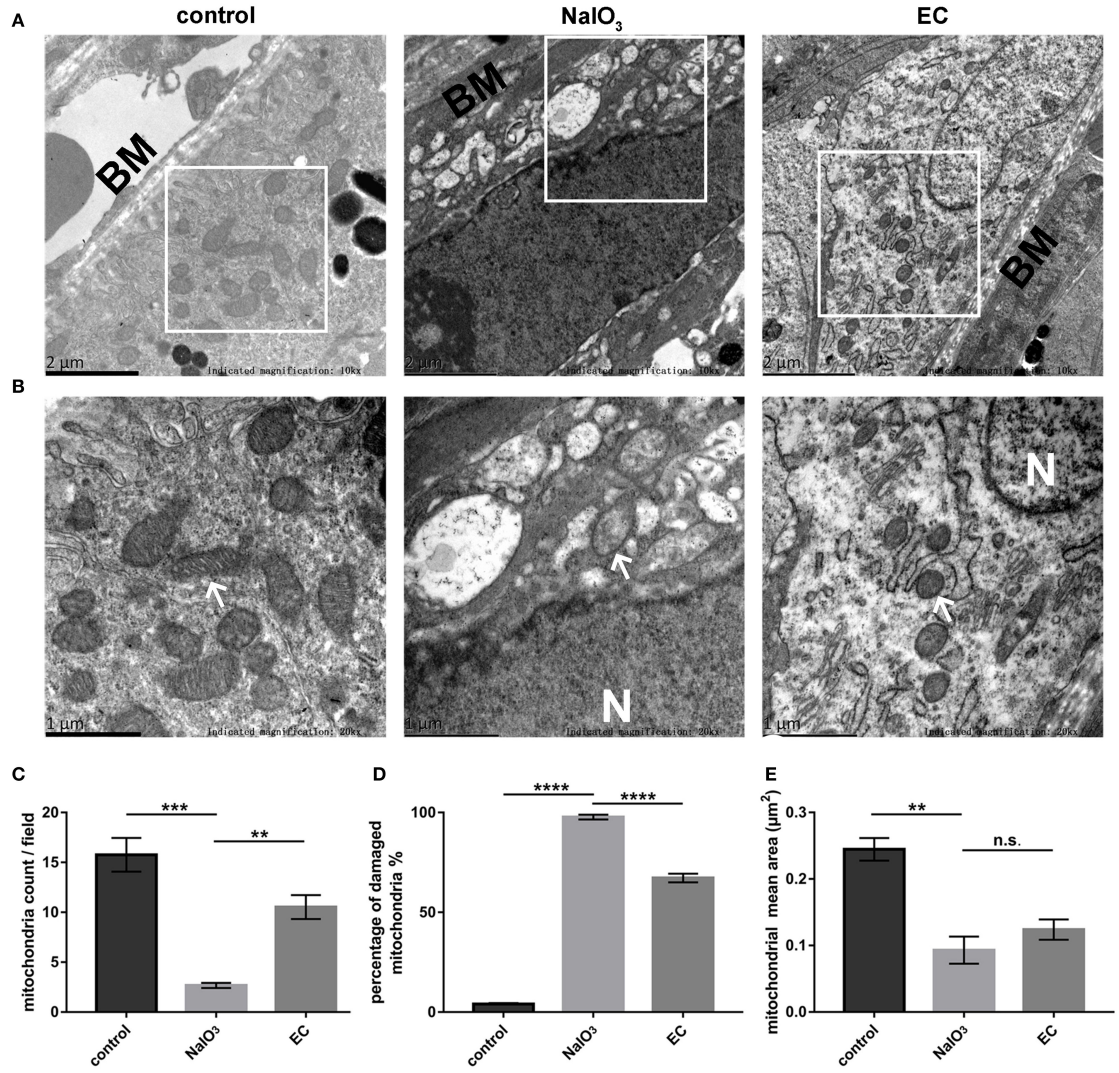
RPE mitochondrial morphological changes detected by TEM 7 days after NaIO_3_ administration. **(A)** and **(B)** Representative TEM micrographs show decreased mitochondria numbers, loss of cristae, and mitochondrial vacuolization in the NaIO_3_ group; EC helped maintain normal mitochondrial morphology. Black BM indicates Bruch's membrane. White arrows indicate mitochondria. White N indicates nucleus. Magnification: 10,000 × (bar = 2 μm) and 20,000 × (bar = 1 μm), respectively. **(C)** Total number of mitochondria per field were increased in the EC group compared to the NaIO_3_ group. **(D)** Percentage of damaged mitochondria per field were decreased in the EC group compared to the NaIO_3_ group. **(E)** There was no apparent difference in size of mitochondria between the NaIO_3_ group and the EC group. Values are presented as mean with SEM; *n* = 4; n.s., no significance. **p < 0.01, ***p < 0.001, ****p < 0.0001.

Ultrastructural analysis verified the protective role of EC in preserving mitochondria in RPE cells. Results obtained by TEM showed abnormal mitochondrial morphology: reduced numbers of mitochondria, extensive disruption of the cristae, and decreased electron density of the matrix. Mitochondrial morphology and structure notably improved with the EC treatment (Figures 7A,B). Although EC did not change the mean area of mitochondria (Figure 7E), it increased the number of mitochondria per field (Figure 7C) and decreased the percentage of damaged mitochondria (Figure 7D) as compared to the NaIO_3_ group.

### EC Modulated the Expression of Proteins Related to Mitochondrial Quality Control

**Figure 8:**
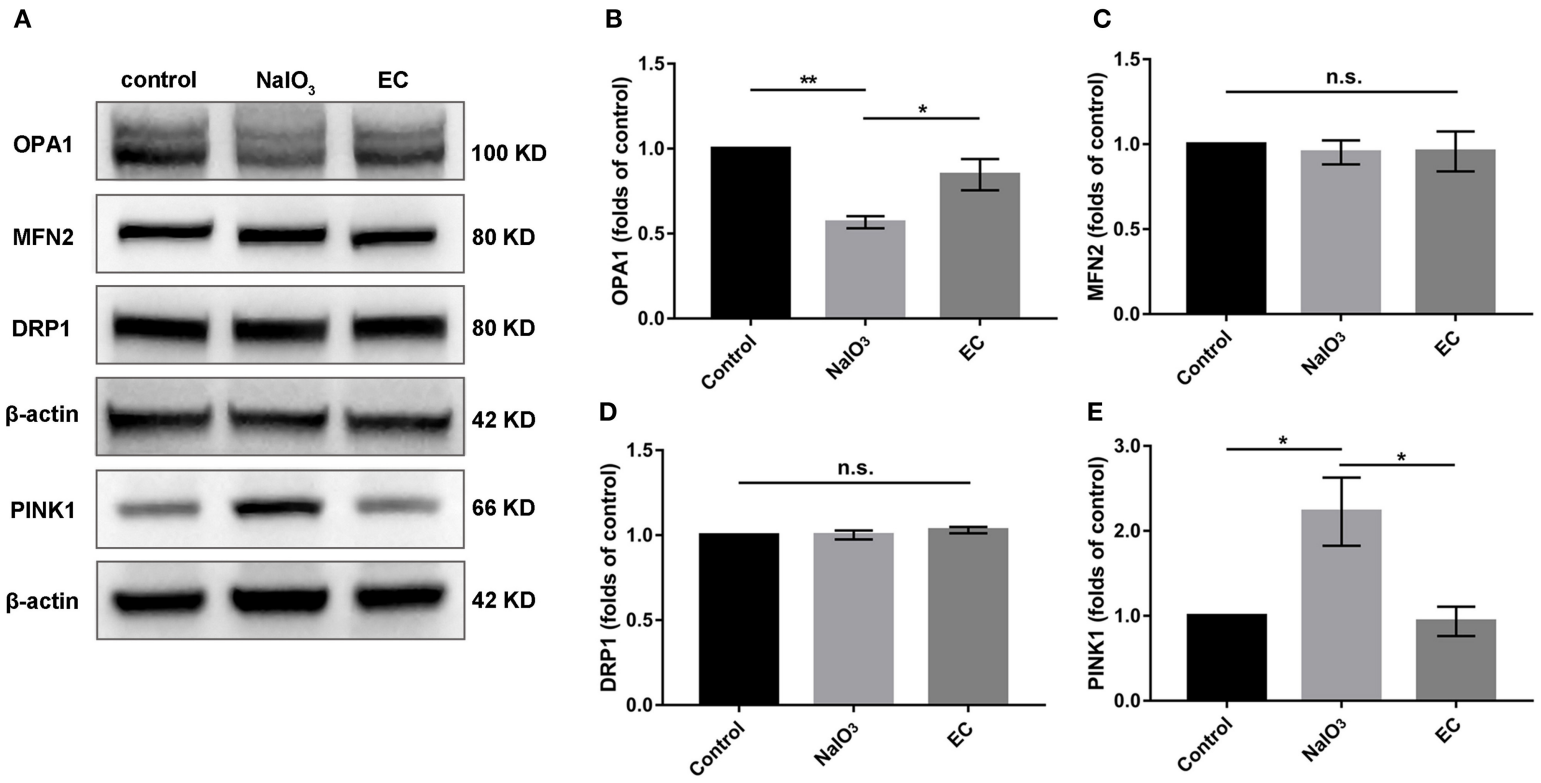
Expression of mitochondrial quality control related proteins after EC and NaIO_3_ co-treatment. **(A)** Key proteins responsible for mitochondrial fission and fusion, and mitophagy-related proteins PINK1 were measured by Western blot. **(B)** Corresponding quantification of the relative expression of OPA1 demonstrated an enhancement with EC treatment, but no significant changes were found in **(C)** MFN2 and **(D)** DRP1. **(E)** Quantification analysis showed that the increase of PINK1 was lower with EC treatment. Values are presented as mean with SEM; *n* = 4; n.s., no significance. *p < 0.05, **p < 0.01.

To determine whether mitochondria-related pathways were involved in the protective effects of EC in NaIO_3_-induced mitochondrial damage, we examined mitochondrial function in retina. First, the expressions of OPA1, MFN2, and DRP1, key proteins responsible for mitochondrial fission and fusion, were measured by Western blot (Figure 8A). The results showed that levels of OPA1, a protein responsible for fusion of inner mitochondrial membranes, were nearly normalized with EC administration (Figure 8B); levels of MFN2 and DRP1 did not change compared to NaIO_3_ injected alone (Figures 8C,D). Interestingly, when treated with NaIO_3_, levels of PINK1 markedly increased; when co-treated with EC, the levels of PINK1 were brought down (Figure 8E).

### EC Regulated Levels of APP, TMEM97, PGRMC1, and Aβ

**Figure 9:**
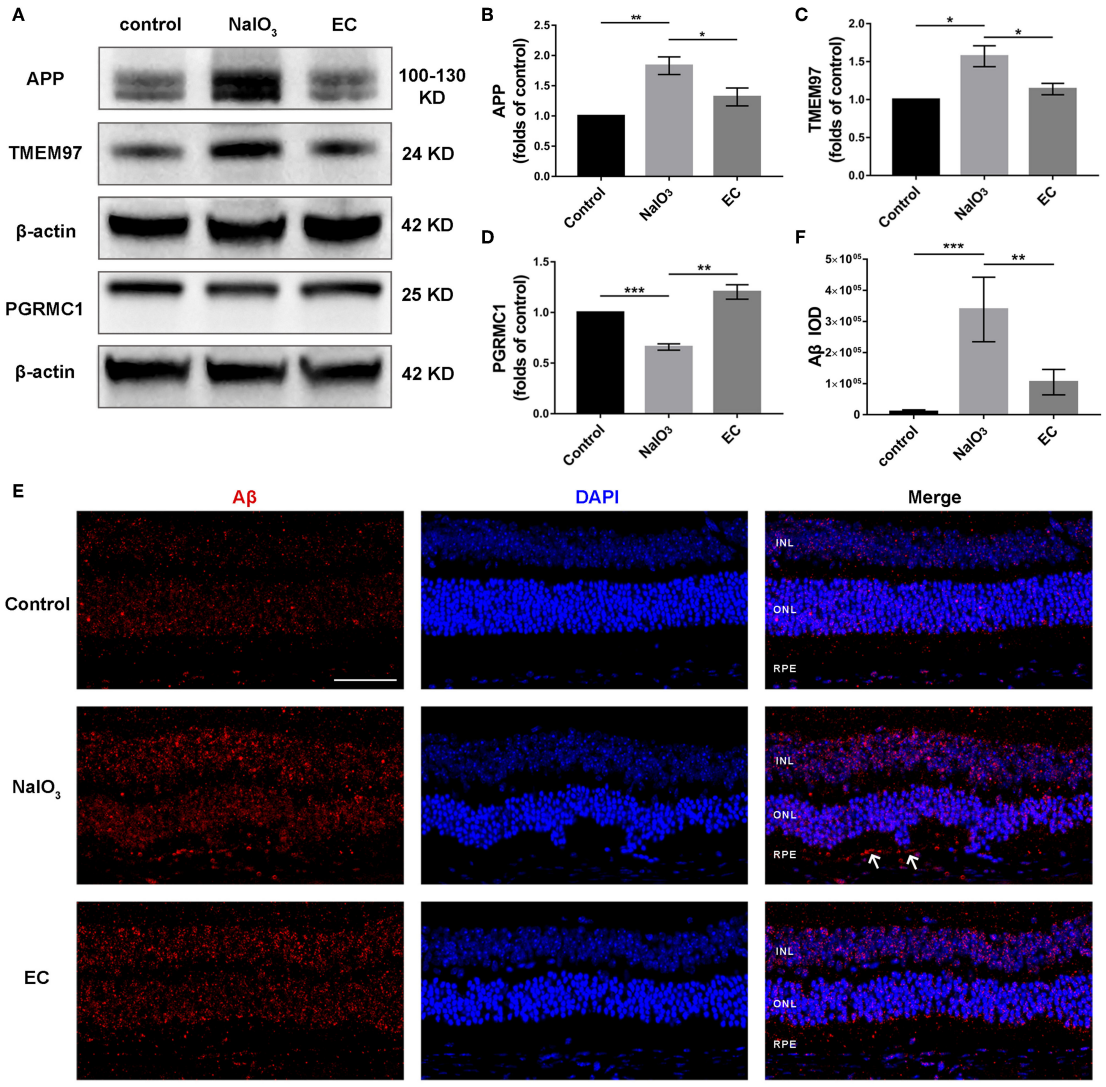
Role of EC on the expression of APP, TMEM97, PGRMC1, and Aβ in retina treated with NaIO_3_. **(A–D)** Representative Western blot images and quantitative analysis of APP, TMEM97, and PGRMC1 levels. **(E)** Representative immunofluorescence images show sub-RPE Aβ accumulation in the three groups. **(F)** Quantification of Aβ fluorescence intensity in the RPE and sub-RPE layer. Aβ (red), DAPI (blue). White arrows indicate sub-RPE Aβ deposits. INL, inner nuclear layer; ONL, outer nuclear layer; RPE, retinal pigment epithelium. Values are presented as mean with SEM; *n* = 3 or 4. *p < 0.05, **p < 0.01, ***p < 0.001.

TMEM97, also known as sigma-2 receptor that important for the neuronal accumulation of Aβ (24, 25), is one of DEGs in AMD detected by bioinformatics analyses, so we explored the role of the TMEM97 and related proteins in this model using Western blot (Figure 9A). Interestingly, we found a notable increase in APP (Figure 9B) and TMEM97 protein levels (Figure 9C) and a decrease in PGRMC1 levels (Figure 9D) 7 days after NaIO_3_ administration. EC treatment lowered this trend significantly. Because the molecular weight of Aβ is too small to be measured by Western blot, we detected its fluorescent intensity using immunofluorescence. The results indicated a trend toward a reduction of sub-RPE Aβ deposits in the EC group (Figures 9E,F).

## Discussion

In this study, we found that EC effectively ameliorated retinal morphology degeneration, protected visual function, and increased mitochondria numbers and quality after NaIO_3_ administration. Here, the TMEM97 protein was identified as a DEG in AMD and found to be elevated in an AMD model for the first time, indicating that the protective effect of EC may be correlated with the involvement of the TMEM97/PGRMC1/Aβ signaling pathway. These results suggest that EC may serve as a potential new approach for the therapy of dry AMD.

Considering there is still inadequate treatment for dry AMD (26), an urgent therapeutic need is required. Flavonoids have received much attention due to their neuroprotective effects in multiple diseases, including degenerative eye diseases (27, 28). More recently, evidence has shown that a food supply of flavonoids was associated with a decreased incidence of AMD in a 15-year population-based cohort study (29). Another study found that flavonoids preserved retinal morphology and functions and reduced the production of reactive oxygen species in an acute model of light-induced retinal degeneration (28). Another flavonoid— epigallocatechin 3-gallate, also exerted its powerful protective effects against cerebral amyloidosis in Alzheimer's transgenic mice (30) and retinal cell death in retinal ischemia-reperfusion mice (31).

EC, a common flavonoid, plays a protective role in central nervous system diseases (17, 18, 32) and diabetic retinopathy (20), but little is known about its neuroprotective effects in degenerative retinal diseases. NaIO_3_ causes necroptosis of the RPE cells and secondary cell death of photoreceptors (33). Therefore, we explored the role of EC in NaIO_3_-induced retinal degeneration—a widely used dry AMD model induced by oxidative stress.

As expected, intravenous injection of NaIO_3_ combined with oral gavage of EC alleviated the main pathological changes, including drusen-like deposits and outer retinal layer thinning, indicating a general protective efficacy of EC for the retina. With the EC treatment, we also observed the visual function recovery of ERG responses and b-wave amplitudes, which were generated from on-and-off bipolar cells. This finding suggests that EC may lead to the protection of neuronal functions in mildly injured bipolar cells.

Bioinformatics analyses provide an efficient and comprehensive method for us to understand molecular mechanisms of complicated diseases. By assessing gene expression profile in normal and AMD retinas, we identified a large amount of DEGs including TMEM97. By carrying out functional enrichment analysis and GSEA, we found that DEGs were closely associated with oxidative stress, astrocyte differentiation, glial cell migration, Aβ clearance, Aβ binding, and mitochondria-related pathways. Those findings served as clues for further experimental exploration and verification.

Glial cells provided strong support to retinal neurons and RPE cells. Under normal conditions, astrocytes and Müller cells express GFAP at detectable levels, and GFAP is dramatically upregulated after damage to the retina (23). The downregulation of GS, a selective marker of Müller cells, is a typical feature of gliosis (23). Our findings that the total fluorescence intensities of GFAP and GS were normalized to some extent with EC treatment suggested that the neuroprotective role of EC may be associated with the blocking of retinal gliosis. This effect was in line with the prevention of glial activation in diabetic retinas treated with EC (20).

Mitochondrial quality control is critical for maintaining mitochondrial homeostasis in high metabolic RPE cells, and failure of any of these processes leads to RPE degeneration (34, 35). Inner mitochondrial protein OPA1 is essential for the fusion of the mitochondrial inner membrane and the maintenance of the cristae structure. With the injection of NaIO_3_, a strong oxidant, we observed a collapse in mitochondrial morphology in RPE cells, along with a sharp reduction in OPA1 levels in retina. In mice treated with exogenous EC, OPA1 protein levels increased. Additionally, mitochondrial numbers and cristae structure were restored, which is consistent with earlier studies (14, 36, 37). Thus, we speculated that EC could improve the dynamic balance of mitochondria, just as had previously been reported (38). PINK1 acts as a checkpoint for mitochondrial quality control systems to maintain mitochondrial homeostasis under normal and damage conditions (39). Our results showing that PINK1 proteins returned to baseline with EC treatment further bolster the possibility that the protective role of EC may be associated with the recovery of mitochondrial functions.

Aβ, an important component of drusen deposits, has long been considered a pathogenic molecule and potential therapy target to prevent or treat AMD (9, 40). Aβ peptides are continuously metabolized by the sequential cleavage of APP, and the overexpression of APP could lead to the accumulation of Aβ (41). Sigma receptors, designated as sigma-1 and sigma-2, represent promising and novel targets for the therapy of retinal diseases (42–47). More recently, TMEM97, a gene that codes for the sigma-2 receptor (24), was identified as new locus for AMD susceptibility (22). TMEM97 combined with PGRMC1 and low-density lipoprotein receptors form a protein complex that is responsible for the cellular uptake of Aβ (25). Sigma-2 receptor antagonist, which destabilizes the Aβ binding site, exerted cognitive enhancing and neuroprotective effects in neurodegeneration (48, 49). Here the subretinal Aβ seemed to be reduced, and protein expression of TMEM97, PRGMC1, and APP returned to near normal levels with EC administration. We demonstrated that TMEM97 and PRGMC1 may be involved in the pathological mechanism of retinal degeneration *via* Aβ accumulation.

There are some limitations in this study. First, we did not validate the protective effects of EC *in vitro*, especially possible molecular mechanisms in cultured RPE cells. The concentration of EC in retina was not detected in this study since it was reported elsewhere (50). Finally, the gene levels of TMEM97 were found to be lower in human AMD retinas while the protein levels of TMEM97 were higher in mice AMD retinas compared to the controls. We speculate this discordance may arise from species (human vs. mice), disease model (AMD vs. NaIO_3_-induced dry AMD model), or duration of disease. We will design a further study to test gene levels of TMEM97 and other DEGs using GSM sets with larger AMD samples, and detect transcription levels and translation levels of those DEGs using molecular experiment.

In conclusion, our results strongly suggest that EC provides powerful protective effects against NaIO_3_-induced retinal degeneration *in vivo*. The mechanisms underlying the activity of EC may be associated with enhanced mitochondrial quality *via* the recovery of fusion and PINK1, along with reduced Aβ accumulation *via* APP, TMEM97 and PGRMC1. The retinal protective effect of EC make it a potential candidate drug for treating dry AMD.

## Data Availability Statement

The raw data supporting the conclusions of this article will be made available by the authors. The datasets presented in this study can be found in online repositories. The names of the repository/repositories and accession number(s) can be found at: https://www.ncbi.nlm.nih.gov/geo/, GSE135092.

## Ethics Statement

The animal study was reviewed and approved by Animal Care Committee of Central South University.

## Author Contributions

MP performed most of the experiments, analyzed the results, generated figures, and wrote the manuscript. XZ and HL helped with designing the experiments. FY helped with performing the experiments and revising the article. XX and WS conceived the designs and revised and approved the manuscript. SX analyzed the data and revised the manuscript. All authors contributed to the article and approved the submitted version.

## Funding

This research was supported by grants from the National Natural Science Foundation of China (Nos. 81974134, 81670858, 81974137, and 82171058), National Key Research and Development Program of China (No. 2020YFC2008205), and Key R&D plan of Hunan Province of China (No. 2020SK2076).

## Conflict of Interest

The authors declare that the research was conducted in the absence of any commercial or financial relationships that could be construed as a potential conflict of interest.

## Publisher's Note

All claims expressed in this article are solely those of the authors and do not necessarily represent those of their affiliated organizations, or those of the publisher, the editors and the reviewers. Any product that may be evaluated in this article, or claim that may be made by its manufacturer, is not guaranteed or endorsed by the publisher.

## Abbreviations

AMD: age-related macular degeneration
APP: amyloid precursor protein
Aβ: amyloid beta
DRP1: dynamin-related protein 1
EC: (–)–epicatechin
ELM: external limiting membrane
ERG: electroretinography
GCL: ganglion cell layer
GFAP: glial fibrillary acidic protein
GS: glutamine synthetase
INL: inner nuclear layer
IS: inner segment
MFN2: mitofusin 2
NaIO_3_: sodium iodate
NES: normalized enrichment score
OCT: optical coherence tomography
ONL: outer nuclear layer
OPA1: optic atrophy 1
OS: outer segment
PGRMC1: progesterone receptor membrane component 1
PINK1: PTEN-induced kinase 1
RPE: retinal pigment epithelium
TEM: transmission electron microscopy
TMEM97: transmembrane Protein 97.
