# Supplementary material for: (–)-Epicatechin Provides Neuroprotection in Sodium Iodate-Induced Retinal Degeneration
Source: Front Med (Lausanne). 2022 Jun 27;9:879901. doi: 10.3389/fmed.2022.879901 (PMC9271623; doi:10.3389/fmed.2022.879901)
Supplement: Supplementary file 3 [file Data_Sheet_3.DOCX]

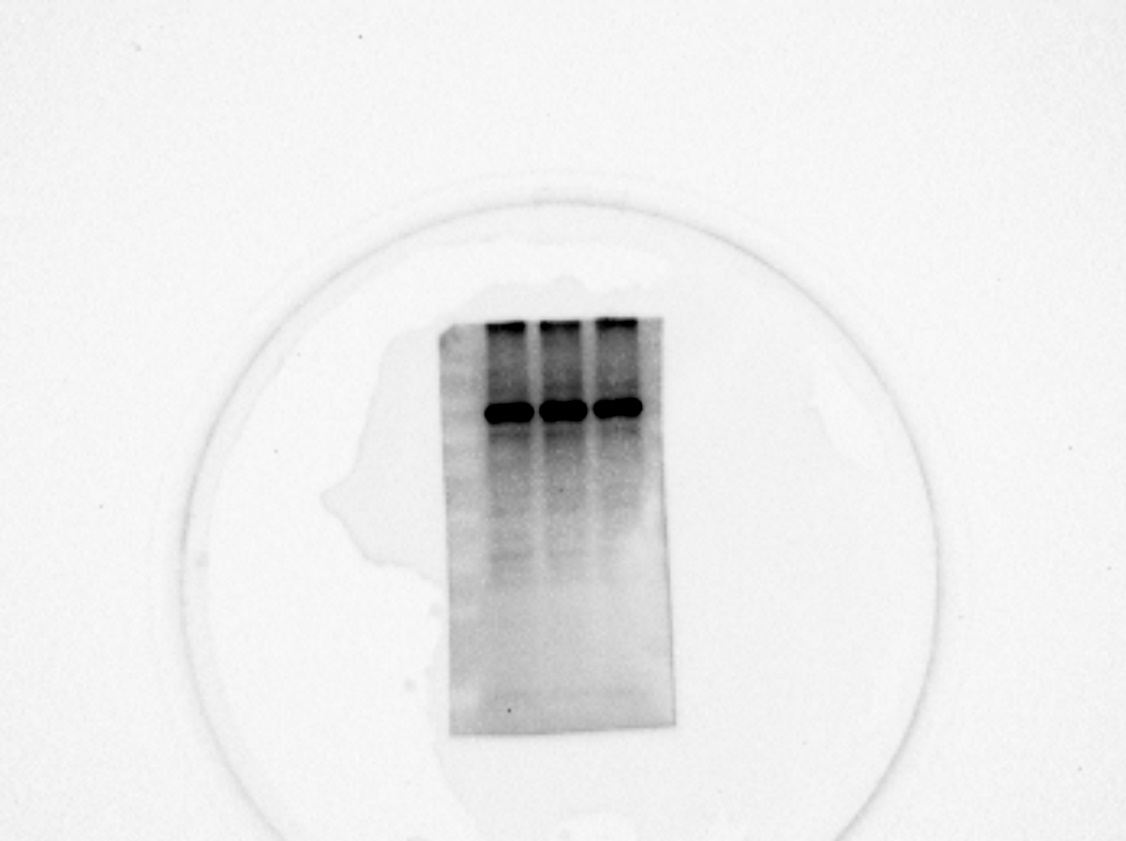


Figure 1. Original Western blot images of MFN2 levels.


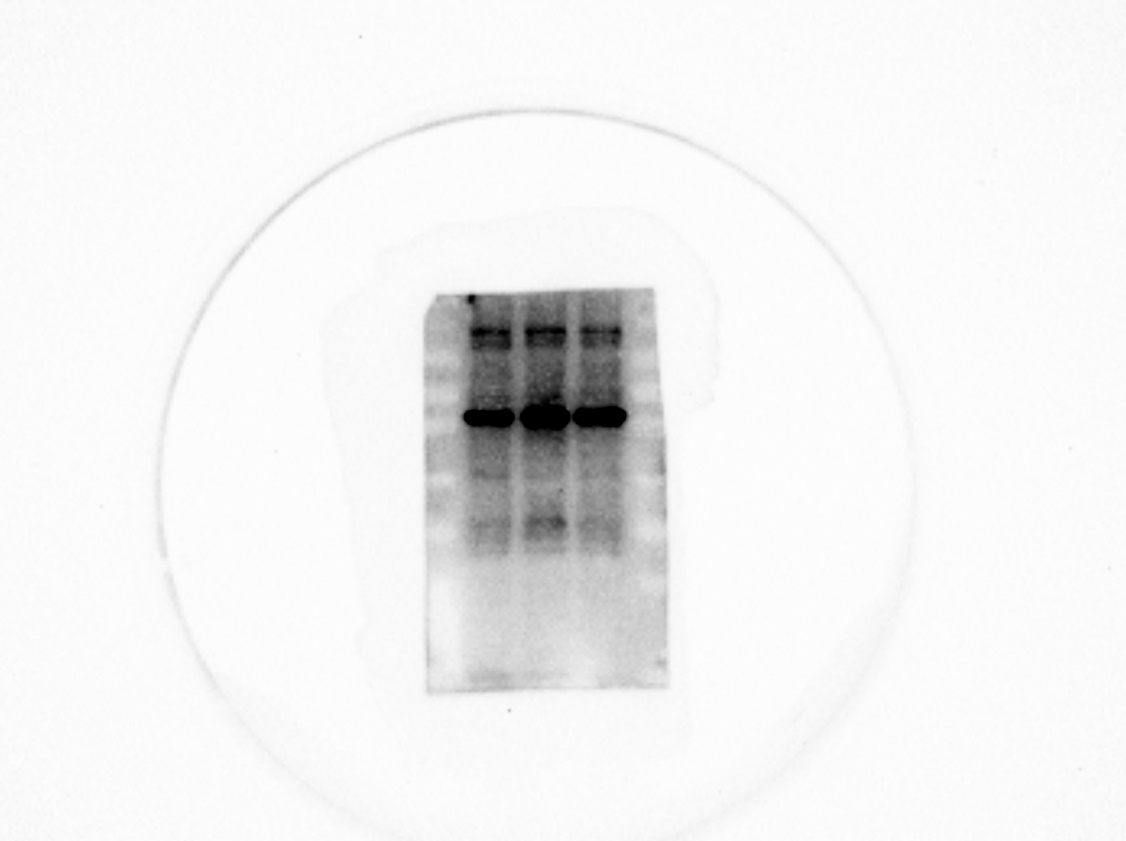


Figure 2. Original Western blot images of PINK1 levels.


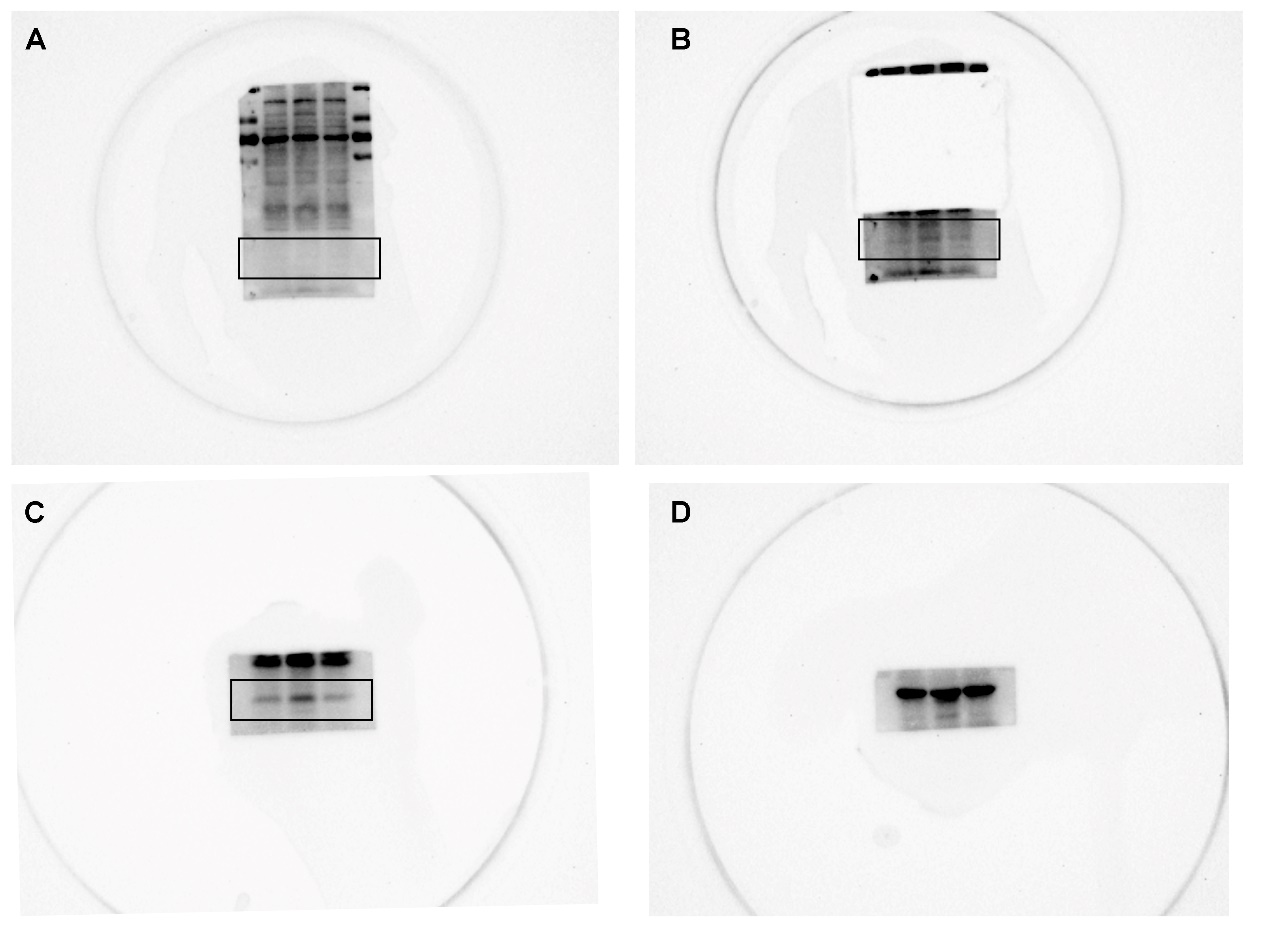


Figure 3. Original Western blot images of TMEM97. (A) Uncropped blot of TMEM97. There are strong nonspecific bands around 70KD, which affect the imaging of TMEM97 (24KD). (B) We used a piece of silver paper to cover the area of nonspecific bands (35KD-180KD), but the bands of TMEM97 are still too weak to observe. We cropped the blot and used the Thermo Scientific™ Restore™ Western Blot Stripping Buffer to remove anti-TMEM97 and secondary antibodies, and re-incubated the same blot with the (C) anti-TMEM97 and (D) the anti-β-actin antibody (which serves as an internal control), respectively. The results show clear bands of TMEM97 around 24KD (although there are still nonspecific bands around 25KD).
